# Wall Slip-Free Viscosity Determination of Filled Rubber Compounds Using Steady-State Shear Measurements

**DOI:** 10.3390/polym15224406

**Published:** 2023-11-14

**Authors:** Dennis Kleinschmidt, Florian Brüning, Jonas Petzke

**Affiliations:** Kunststofftechnik Polymer Engineering Paderborn, Faculty of Mechanical Engineering, Paderborn University, 33098 Paderborn, Germany; florian.bruening@ktp.uni-paderborn.de (F.B.); jonas.petzke@ktp.uni-paderborn.de (J.P.)

**Keywords:** rheology, closed cavity rheometer, steady-state shear viscosity, rubber, wall slip, Cox–Merz rule

## Abstract

The high-pressure capillary rheometer (HPCR) represents a state-of-the-art instrument for the determination of rheological properties for plastics and rubber compounds. Rubber compounds have an increased tendency to exhibit flow anomalies depending on the compound ingredients and the processing parameters. Combined with non-isothermal effects due to dissipative material heating, this causes rheological material measurements and the resulting material parameters derived from them to be affected by errors, since the fundamental analytical and numerical calculation approaches assume isothermal flow and wall adhesion. In this paper, the applicability of the empirical rheological transfer function of the Cox–Merz rule, which establishes a relationship between shear viscosity measured with a HPCR and complex viscosity measured with a closed cavity rheometer (CCR), is investigated. The Cox–Merz relation could not be verified for an unfilled EPDM raw polymer or for filled, practical rubber compounds. Using a closed cavity rheometer, a methodology based on ramp tests is then introduced to collect wall slip-free steady-state shear viscosity data under isothermal conditions. The generated data show high agreement with corrected viscosity data generated using the HPCR, while requiring less measurement effort.

## 1. Introduction

The continuous development of rubber compound formulations to ensure increasing demands for application-specific product properties requires accurate knowledge of material properties to predict the process and processing behavior. In this context, analytical and numerical simulation tools are used to reduce development times and minimize sources of error. In this respect, rheological material properties are of central importance and are conventionally determined using high-pressure capillary rheometers (HPCRs). Compared to commercial plastics, analytical and numerical process design approaches more often fail for rubber compounds. Due to non-isothermal effects and wall slip, rheological material measurements and the material parameters derived from them are affected by errors, since the fundamental analytical and numerical calculation approaches assume isothermal flow and wall adhesion [1,2,3,4]. Therefore, a better understanding of the rheological behavior of filled rubber compounds and the availability of suitable rheological characterization methods is essential.

The rubber process analyzer (RPA), technically a type of closed cavity rheometer (CCR), is a standard test instrument in the rubber processing industry. In addition to determining crosslinking kinetics, the RPA can be used to analyze the viscoelastic material behavior and the flow behavior of filled rubber compounds. The flow behavior is characterized by the complex viscosity, which is described by frequency sweeps with stepwise variation of the frequency at constant strain and temperature [5,6].

There are empirical rheological transfer functions in the literature that relate the rheological parameters of shear viscosity from HPCR measurements to the complex viscosity from CCR measurements. This includes the Cox–Merz relation and the Gleißle mirror relations. In principle, it is not possible to physically derive these correlations because they are based on experimental results. Nevertheless, these empirical transfer functions are valid for a large number of thermoplastic melts, where the limits of transferability are mainly attributed to wall slip and non-isothermal effects [3,7,8,9].

In addition to the ability to characterize the flow behavior using frequency sweeps, ramp tests allow for the determination of steady-state shear viscosity. A ramp test is a measurement method in which a defined strain is accelerated uniformly over a defined time interval, thereby inducing a shear rate in the material [3].

By definition, RPA measurements are considered free of wall slip, and, unlike the HPCR, the RPA does not require time-consuming correction procedures to determine the true viscosity data. Combined with the fact that RPA measurements can be performed on small amounts of the material under investigation, this means that RPA studies provide an alternative for describing the flow behavior of filled rubber compounds [1,10,11].

The objective of this research is to verify the validity and thus the applicability of the Cox–Merz rule as an empirical rheological transfer function for practical EPDM rubber compounds exhibiting both adhesive and slip properties. In addition, the capabilities and limitations of steady-state shear viscosity determination using a closed cavity rheometer (rubber process analyzer) as an alternative to conventional high-pressure capillary rheometry are presented. The question addressed is whether this method is a time-efficient alternative to obtain true viscosity data that correlates with the time-consuming corrected viscosity data obtained using an HPCR.

### 1.1. High-Pressure Capillary Rheometry

The high-pressure capillary rheometer is widely used to characterize the flow behavior of thermoplastic melts and rubber compounds. With this instrument, it is possible to determine the viscosity over the process-relevant shear rate range. The determination of viscosity as a rheological parameter requires knowledge of the shear rate and the shear stress at the capillary wall. Compared to round hole nozzles, the use of slit capillaries allows more direct pressure and temperature measurements along the flow direction in the slit capillary, which significantly reduces the measurement effort and allows run-in effects to be neglected [3,12].

For the derivation of the viscosity relation between wall shear stress and shear rate, the following fundamental assumptions are made [13]:

- Fully developed, steady, laminar flow (Newtonian flow behavior)
- No non-isothermal effects
- Incompressible fluid with no pressure dependence of viscosity
- Wall adhesion

The apparent wall shear rate ${\dot{\gamma }}_{app}$ in a slit capillary of width W and heigth H depends on the total volume flow ${\dot{V}}_{total}$ that is passed through the slit capillary. This relationship, given in Equation (1), was derived by assuming a wall-adhering Newtonian fluid [3,12]:

(1) $${\dot{\gamma }}_{app}=\frac{6\times {\dot{V}}_{total}}{W\times H^{2}}$$

${\dot{\gamma }}_{app}$: Apparent shear rate
${\dot{V}}_{total}$: Total volume flow
W: Width of the slit capillary
H: Height of the slit capillary

The occurrent wall shear stress $\tau_{w}$ in the slit capillary is determined by measuring the pressure drop ∆p over the distance of the pressure measuring points ∆L. This is done under the assumption that the width W is much greater than the height H of the capillary to avoid edge effects, as shown in Equation (2) [3]:

(2) $$\tau_{w}=\frac{H}{2}\times \frac{∆p}{∆L}$$

$\tau_{w}$: Wall shear stress
∆p: Pressure drop in the slit capillary
∆L: Distance between pressure measuring points

**Figure 1:**
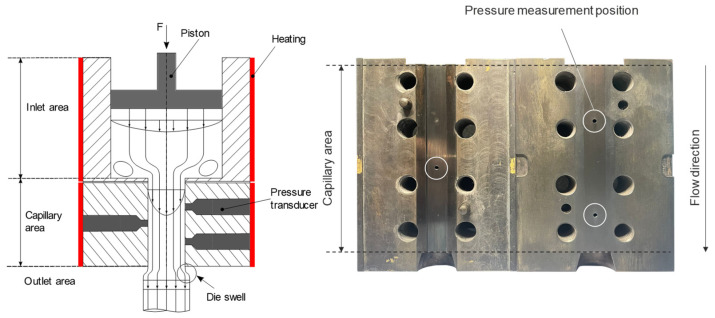
Principle diagram of a high-pressure capillary rheometer using a slit capillary.

The schematic design of the high-pressure capillary rheometer is shown in Figure 1.

For the description of the shear-thinning flow behavior of rubber compounds, it is sufficient to apply the power law expressed in Equation (3), as Leblanc et al. [14] could not detect a viscosity plateau for a variety of rubber polymers using diverse rheometer designs over a broad range of shear rates $\dot{\gamma }$ from 10^−5^ s^−1^ to 10^4^ s^−1^. In this context, the power law with the consistency factor *K* and the flow law exponent *n* describes the viscosity *η* of rubber compounds according to Ostwald and de Waele in the following form [3,14,15]:

(3) $$\eta =K\times {\dot{\gamma }}^{n-1}$$

η: Viscosity
K: Consistency factor
$\dot{\gamma }$: Shear rate
n: Flow exponent

The assumptions used to derive the introduced functions are not fully valid for various types of complex fluids such as polymer melts and solutions, as well as rubber compounds [16]. Accordingly, complex correction procedures are required to obtain the true viscosity data. As the shear rate increases, both shear friction and shear heating increase. Due to the poor thermal conductivity of rubber compounds in particular, heat accumulation and temperature rise in the material are initiated. This was demonstrated by Hornig using filled FKM compounds [17].

Wall slip leads to an incorrect determination of shear stresses and other material properties. When wall slip effects occur during processing, they cannot be minimized or prevented without affecting the process. As a result, it is necessary to evaluate the wall slip behavior specifically for the material and to take it into account in mathematical modeling and simulations [18]. For the mathematical quantification of wall slip velocities in rheological measurements, different approaches exist for different types of rheometers. In particular, the Mooney method [19], as well as the modified Mooney method by Geiger [20], have been used in recent work to determine slip velocities. With respect to slit capillaries, the Mooney and Geiger correction methods require data from at least two slit capillaries with the same W/H and L/H ratios, which significantly increases the time required for the measurement. For a review of the main studies on wall slip effects focusing on filled rubber compounds, refer to [2], while Hatzikiriakos [16] has given a comprehensive overview of wall slip mechanisms in complex fluids in general.

**Figure 2:**
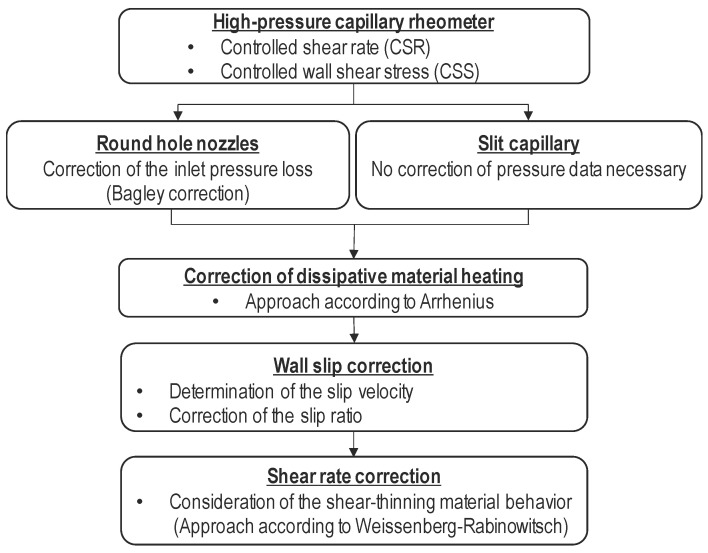
Correction procedure in rheological investigations using the HPCR.

In addition to dissipative material heating and wall slip effects, rubber compounds and polymer melts are shear-thinning, non-Newtonian fluids, so Equation (1) is not generally applicable to rubber compounds and polymer melts. The true shear rate is determined using the Rabinowitsch correction [21] based on the apparent shear rate. Figure 2 illustrates the workflow of a rheological study using the HPCR, including the associated correction procedures.

### 1.2. Rubber Process Analyzer

In the rubber processing industry, the rubber process analyzer (RPA) represents a closed cavity rheometer (CCR) from a process engineering perspective. Due to the variable setting of the parameters (amplitude, frequency), it offers a wide range of applications for the characterization of viscoelastic materials for quality and incoming goods inspection as well as for research and development. For example, the rubber process analyzer is used to evaluate flow behavior and to analyze the product quality of elastomers. Another important application is the determination of the crosslinking kinetics of rubber compounds [5].

**Figure 3:**
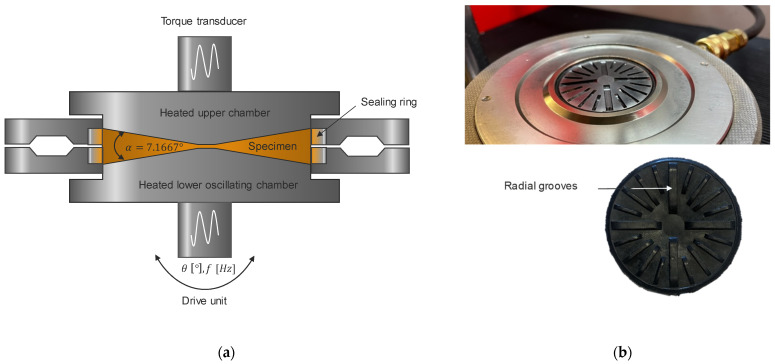
(**a**) Schematic illustration of the RPA in accordance with [23]; (**b**) test chamber and specimen with radial grooves.

The test chamber of the rubber process analyzer includes two sealed, heated, and biconically designed test-chamber halves (refer to Figure 3a). The cone–cone configuration with a cone angle of 7.167° of the D-RPA 3000 from MonTech Werkstoff-Prüfmaschinen GmbH (Buchen, Germany) results in a nearly homogeneous shear rate field in the specimen to be analyzed. The lower half of the test chamber oscillates at a specified deflection angle and frequency, while the torque required for deformation is recorded in the upper half of the test chamber. The test-chamber halves are also characterized by radial grooves for torque transmission, which, in combination with the applied test-chamber closing pressure, allow for the elimination of wall slip effects (see Figure 3b) [3,11,12,22].

The results obtained include the elastic components of the specimen in the form of the storage modulus $G^{\prime }$ and the viscous components in the form of the loss modulus $G^{\prime \prime }$. The storage modulus $G^{\prime }$ is a measure of the elastic component of the material and is proportional to the proportion of the energy that can be recovered upon relaxation, while the loss modulus $G^{\prime \prime }$ is a measure of the viscous component of the material and is proportional to the proportion of the deformation energy that is dissipated as heat. The differentiation is made based on the phase shift between specimen excitation and response. The behavior of viscoelastic materials such as rubber compounds is intermediate between the ideal cases of ideal elastic and ideal viscous material behavior [3,6].

#### Characterization of Viscoelastic Material Behavior

Amplitude sweeps are dynamic oscillatory material measurements in which the deflection angle (strain) is varied in discrete steps at constant frequency and temperature [6]. In this context, SAOS (small-amplitude oscillatory shear) and LAOS (large-amplitude oscillatory shear) determinations are methodologies that are becoming increasingly important in the field of rubber rheology. The deflection angle θ specified in the dynamic investigations can be converted into a percentage strain γ using the double cone angle α of the biconical test chamber, as represented by Equation (4) [6,13]:

(4) $$\gamma =\frac{\theta }{\alpha }=\frac{\theta }{7.1667{}^{\circ}}$$

γ: Strain
θ: Deflection angle
α: Double cone angle of the biconical test chamber

**Figure 4:**
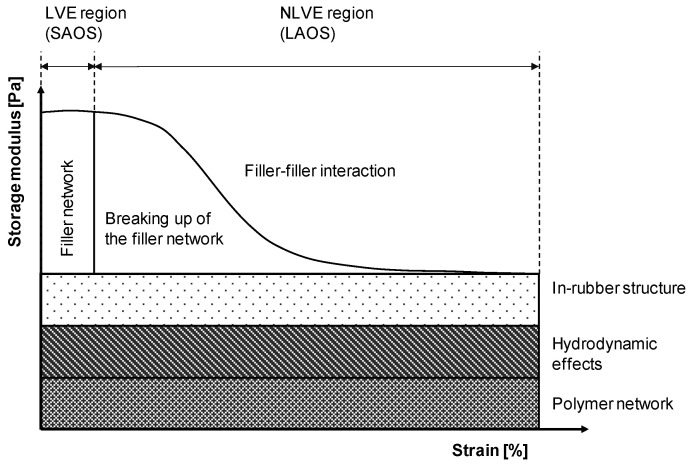
Characterization of the Payne effect using an amplitude sweep in accordance with [25].

In the linear viscoelastic region, the storage and loss modules $G^{\prime }$ and $G^{\prime \prime }$ have a constant plateau value. Using an amplitude sweep, it is possible to determine the material-specific linear viscoelastic (LVE) region, as well as its limit [6]. Typically, in filled systems, the storage modulus results are high at low strains and decrease when a larger strain amplitude is applied to the specimen (nonlinear viscoelastic region (NVLE)). This phenomenon was fundamentally studied by Payne [24] and is called the Payne effect in the literature, describing the breakdown of the mechanically unstable filler network with increasing strain. It is visualized in Figure 4 [5]. According to the Payne model, the resulting dynamic modulus is composed of the contributions of the polymer network, the hydrodynamic reinforcement, and the filler–polymer as well as filler–filler interactions (both the intact and the degraded filler networks) [6,25,26,27].

Frequency sweeps are dynamic oscillation measurements in which the frequency is varied in discrete steps at constant amplitude (strain) and temperature. Since the frequency is the inverse of time, a frequency sweep describes the time-dependent deformation behavior of the specimen. In addition to the determination of rheological parameters for the elastic and viscous material behavior, the frequency dependence of the modules allows the determination of relaxation time spectra as well as conclusions about the molecular structure of the material. Thus, the intersection of the storage and loss modules is a measure of the chain branching as well as the mean molar mass and molar mass distribution of the investigated material [6,12,28].

### 1.3. Empirical Rheological Transfer Functions

A number of empirical relationships exist in the literature to relate the rheological parameters of oscillatory shear flow to those of steady-state shear flow. Their derivation is based on experimental investigations, for which a theoretical derivation is usually not possible. In addition to Gleißle mirror relations I and II, the Cox–Merz rule is one of the most important empirical rheological transfer functions, with considerable practical importance in the plastics industry [8].

#### 1.3.1. Cox–Merz Rule

In the linear viscoelastic (LVE) region, the complex viscosity can be accessed by applying a small-amplitude oscillatory shear flow (SAOS). The magnitude of the complex viscosity is composed of the parts of the components of the storage modulus $G^{\prime }$ and the loss modulus $G^{\prime \prime }$, as given by Equation (5) [3,11]:

(5) $$\left|\eta^{*}\left(\omega \right)\right|=\frac{\sqrt{{G^{\prime }}^{2}+{G^{\prime \prime }}^{2}}}{\omega }$$

$\eta^{*}$: Complex viscosity
$G^{\prime }$: Storage modulus
$G^{\prime \prime }$: Loss modulus

The complex viscosity from dynamic measurements in the LVE region is related to the viscosity η measured at steady-state shear flow by the empirical Cox–Merz rule. The magnitude of the complex viscosity and the steady-state shear viscosity have the same value when the angular frequency ω and the apparent shear rate ${\dot{\gamma }}_{app}$ are identical, leading to Equation (6) [3,29]:

(6) $$\left|\eta^{*}\left(\omega \right)\right|=\eta \left({\dot{\gamma }}_{app}=2\times \pi \times f=\omega \right)$$

f: Frequency

As claimed by Cox and Merz, a correction of the apparent shear rate according to the Weissenberg–Rabinowitsch correction for the polystyrene types with different molar masses used in the investigations does not significantly affect the results; therefore, Equation (7) is valid: [29]:

(7) $$\left|\eta^{*}\left(\omega \right)\right|=\eta \left(\dot{\gamma }=\omega \right)$$

An overview of theoretical considerations for the derivation of the Cox–Merz rule as well as approaches to modify the model function is given by Ansahl et al. [7].

The applicability of the Cox–Merz relationship has been demonstrated experimentally for a variety of polymer melts [3,7,11,22,30]. A detailed investigation of the validity of the Cox–Merz relationship for polyolefin melts is provided by Snijkers and Vlassopoulos [31]. In addition to thermoplastics, the empirical model function from Cox and Merz has also been demonstrated for various rubber raw polymers such as EPDM [32,33], NBR [34], HNBR with different molecular weights, and FKM [22]. The main advantage of the Cox–Merz rule is the prediction of shear viscosity based on dynamic data [7].

Since the Cox–Merz rule represents an empirical relationship between the complex viscosity from dynamic frequency sweeps and the steady-state shear viscosity, a closed theoretical derivation of this relationship is not possible. The erroneous transferability between viscosity curves from oscillatory rheological and capillary rheometric studies has been attributed in the literature to various causes in the determination of steady-state shear viscosity and complex viscosity [7,9].

- Viscous material heating
- Pressure dependence of viscosity
- Wall slip effects

Using blends of natural rubber (NR) and chlorinated polyethylene (CPE), Phewthongin et al. [35] showed that the correlation between viscosity curves obtained by oscillatory rheology and capillary rheometry can only be obtained when the data from high-pressure capillary rheometry using round hole nozzles are corrected. In addition to the correction for the inlet pressure loss (Bagley correction), the consideration of the shear-thinning flow behavior (Weissenberg–Rabinowitsch correction) is also necessary. Hornig and Kielmann [22] showed this for filled HNBR compounds with the addition that the slip component (Mooney correction) had to be taken into account due to wall slip effects.

Yang and Li [9,36] investigated the wall slip behavior of a filled rubber compound by comparing viscosity data obtained from oscillatory shear and steady-state shear. The apparent failure of the empirical Cox–Merz relationship [29], which states that shear viscosity is identical to complex viscosity at appropriate shear rates and angular frequencies, is explained by the occurrence of wall slip effects. This is in agreement with the studies of Ansari et al. [7].

Ellwanger et al. [11] attribute the deviations of the Cox–Merz relationship for thermoplastic melts to wall slip effects in the determination of the steady-state shear viscosity due to a change in the slope of the shear-thinning viscosity range (change in the flow exponent). Due to the potential influencing variables, Ellwanger et al. [11] assume that the empirical relationship is valid within a 15% deviation between the complex viscosity and the steady-state shear viscosity.

Another reason for possible discrepancies between the rheological parameters is related to the underlying flow types. The determination of the complex viscosity is based on dynamic frequency measurements in which the material sample is subjected to oscillating shear flow. In addition, no mass transfer takes place during these frequency sweeps, so a constant material sample is sheared. In shear viscosity determination using high-pressure capillary rheometers, a laminar, stationary pressure flow is the initial condition. In addition, a continuous mass transfer occurs in these rheological studies [37,38].

The basic requirement for the validity of the Cox–Merz rule is the performance of oscillatory rheological studies in the LVE region (SAOS) [39]. In filled systems, such as rubber compounds, the LVE region is shifted to low strain amplitudes, resulting in the limiting case that the LVE region is outside the measurement range of the rheometer and cannot be characterized. This results in the need to perform dynamic frequency measurements in the nonlinear viscoelastic region.

Due to the above-mentioned factors and limitations, the Cox–Merz rule is not universally applicable and needs to be validated for unknown materials and material systems.

To ensure the transferability of the Cox–Merz rule, Gleißle and Hochstein [39] presented a generalized form of the Cox–Merz rule to shift the values from high-pressure capillary rheometry and the values from oscillatory rheometry to each other by means of an empirical shift factor, $k_{v}$, represented by Equation (8) [39]:

(8) $$\left|\eta^{*}\left(\omega \right)\right|=\frac{\eta \left(k_{v}\times \dot{\gamma }=\omega \right)}{k_{v}}=\frac{\left|\eta^{*}\left(\omega \right)\right|}{k_{v}}$$

$k_{v}$: Shift factor

The shift factor $k_{v}$ takes values greater than or equal to one. When the displacement factor $k_{v}$ has a value of one, the generalized form of the Cox–Merz rule corresponds to the original formulation. The determination of the material-specific displacement factor requires investigations with the high-pressure capillary rheometer as well as the oscillatory rheometer, which results in a time-consuming experimental determination and a limited applicability to other material systems.

#### 1.3.2. Ramp Test

In addition to dynamic measurements, modern RPAs also provide access to the transient (time-dependent) viscosity and the steady-state shear viscosity using ramp tests ([32], p. 42). A ramp test, also known as a stress test, is a test method in which a defined strain is applied at a uniformly accelerated rate over a defined time interval. A shear rate is induced by the selection of the strain and the time at which the strain is applied. With respect to the constructive design of the rubber process analyzer, an approximately constant shear rate field is generated due to the biconical test-chamber design [3].

In the ramp test, the torque is measured as a function of time, resulting in a division into a time-dependent (transient) viscosity curve and a time-independent (steady-state) viscosity curve. This allows access to the shear stress and thus to the transient viscosity $\eta^{+}(t_{s})$ as a function of shear time, given by Equation (9) [3]:

(9) $$\eta^{+}\left(t_{s},\dot{\gamma }\right)=\frac{\tau_{edge}\left(t_{s},\dot{\gamma }\right)}{\dot{\gamma }}$$

$\eta^{+}$: Transient viscosity
$t_{s}$: Shear time
$\tau_{edge}$: Edge shear stress

The transient viscosity shows a larger overshoot with increasing shear rate. This is followed by the steady-state shear viscosity. The envelope of all curves is the shear-rate-independent linear viscoelastic stress viscosity ${\eta_{0}}^{+}(t_{s})$ ([3], p. 73f):

The linear viscoelastic stress viscosity ${\eta_{0}}^{+}(t_{s})$ and the shear-thinning viscosity drop $\eta (\dot{\gamma })$ are identical in numerical value if the shear rate $\dot{\gamma }$ is equal to the reciprocal of the shear time $t_{s}$. This relation is called mirror relation I and is shown in Equation (10) [3]:

(10) $$\eta ={\eta_{0}}^{+}\left(t_{s}=\frac{1}{\dot{\gamma }}\right)$$

$\;{\eta_{0}}^{+}$: Linear viscoelastic stress viscosity

Regardless of the validity of the mirror relation I, the steady-state viscosity function $\eta (\dot{\gamma })$ contains the viscosity values from the steady-state region and thus the values that occur after a sufficiently long shear period [3].

The main problem of conventional RPA measurement is the limitation of the maximum deflection to 360°. Depending on the device, only shear rates in the range of 0.1 to 30 s^−1^ can be achieved [32]. The limitation of the maximum deflection leads to the fact that, depending on the shear rate and the material to be investigated, only the transient and not the steady-state shear viscosity can be described [33,40].

To date, only a limited number of publications have been published on the determination of steady-state shear viscosity using closed cavity rheometers. The first studies were performed by White et al. [41] in 1991 using rotational rheometers on SBR. Buhrin et al. [1,10,32] investigated the method of ramp tests on commercial EPDM raw polymer, EPDM compounds, and a truck tire tread compound with focus on wall slip effects by varying of the test-chamber geometry [32]. Heyer et al. [40] performed ramp tests on NR and EPDM raw polymers as well as an oil-containing FKM compound and compared the data with those of the Cox–Merz rule from frequency sweeps, while Ellwanger et al. [11] validated the methodology of ramp tests on thermoplastics (LLDPE, LDPE, PBD) with respect to comparability with the HPCR.

In principle, the RPA is assumed to be free of corrections [32]. The combination of a high internal chamber pressure and the use of test chambers with radial grooves leads to the fact that wall slip effects are unbound [1,10,11,32]. Compared to capillary rheology, no time-consuming and error-prone correction procedures are required. Furthermore, when using the RPA, no time-consuming cleaning steps are necessary, provided that a release film is used for the measurements [32]. In addition, the reproducibility of the results with RPA is reported to be high and more pronounced than with HPCR [32]. The closed cavity also avoids the edge breakage that occurs in commercial open-cavity rheometers due to a lack of melt elasticity [11].

## 2. Materials and Methods

### 2.1. Investigated Materials

A sulfur-crosslinking ethylene–propylene–diene rubber compound based on a Keltan 6950 C according to ISO 4097 [42] was used for the rheological investigations to ensure high relevance for the technical rubber industry. Based on this compound formulation, another EPDM rubber compound was developed and produced that differs significantly in terms of filler content and filler type. Compared to the EPDM compound Pr. 3, the carbon black content was reduced, and silica was added, since silica is an important filler, especially in the tire industry. The composition of the compounds is shown in Table 1.

Both the Cox–Merz rule and the ramp test method have been demonstrated in the literature for various thermoplastic melts. In order to prove the applicability of the Cox–Merz rule and the ramp test method to the RPA used in this work, preliminary tests were carried out on a non-additivated, low-density polyethylene (Lupolen 1840 D) from LyondellBasell (Rotterdam, Netherlands) that is used in blown film extrusion.

### 2.2. High-Pressure Capillary Rheometer

The conventional characterization of the flow behavior of the presented materials was performed on a Rheograph 50 high-pressure capillary rheometer from Göttfert Werkstoff-Prüfmaschinen GmbH (Buchen, Germany) using a slit capillary (width 10 mm, height 1 mm, length 100 mm). Three equidistant pressure transducers ($P_{in},P_{mid}$,$\;P_{out}$) were used along the length of the slit capillary to determine the normal pressure orthogonal to the flow direction. A shear rate range from 1 to 1000 s^−1^ was considered, and measurements were made from high to low shear rates. A detailed description of the rheological investigations by means of HPCR measurement as well as the associated corrections is given in [2], and is therefore omitted at this point. In the following, the fully corrected true viscosity data from the HPCR study series are considered.

### 2.3. Closed Cavity Rheometer (Rubber Process Analyzer)

In order to characterize the viscoelastic material behavior of the filled rubber compounds, extensive investigations were carried out on a D-RPA 3000 from MonTech Werkstoff-Prüfmaschinen GmbH (Buchen, Germany). The rubber process analyzer is an oscillating rheometer, the basic design and operating principle of which are described in Section 1.2. The drive unit is designed to allow for both oscillatory motion as well as continuous rotation without angle limitation. The main specifications of the RPA used are listed in Table 2.

### 2.4. Investigation Plan and Evaluation Methods

#### 2.4.1. Amplitude Sweep

The amplitude sweep, defined in Table 3, is used to determine the linear viscoelastic range and to describe the Payne effect. Since the specimen is placed in a test chamber heated to the material-specific processing temperature under ambient conditions, a sufficient heating phase without specimen deformation (preconditioning) must be provided prior the test to ensure a homogeneous temperature profile in the specimen. The occurrence of specimen deformation during the heating phase of the amplitude sweep should be avoided, as it will affect the Payne effect. To reduce the contamination of the test chamber and the cleaning effort required during oscillatory measurements, polyamide release films with a film thickness of 25 µm are used on both sides of the specimen. In preliminary studies, the influence of the use of test films on the measurement results was analyzed for the EPDM compounds under investigation. Irrespective of the material and temperature, the deviation between measurements with and without test film was less than 5% on average, so the effect of the test film on the measurement results is considered negligible.

To determine the limit value of the linear viscoelastic range, DIN 53019 4 specifies a maximum permissible deviation of 5% from the plateau value of the storage, loss, or complex modulus [43]. The relationship shown in Equation (11), which was developed in preliminary investigations, is suitable for describing the deformation-dependent storage and the loss modulus of filled rubber compounds:

(11) $$G\left(\gamma \right)=\frac{a_{1}}{{\left(1+a_{2}\times \gamma \right)}^{a_{3}}+a_{4}\times \gamma }$$

$a_{1}$: Zero modulus
$a_{2}$: Transition strain
$a_{3}$: Filler network fracture exponent
$a_{4}$: Diffraction coefficient

Using Equation (11), considering the maximum deviation of 5% of the plateau value γ provides the amplitude limit value of the linear viscoelastic range $\gamma_{crit}$. These boundary conditions must be observed for both the storage module and the loss modulus. To determine the LVE region that is valid for the entire frequency range of the frequency sweep, the amplitude sweeps were performed at two frequencies approximately covering the frequency range of the frequency sweep. Amplitude sweeps with frequencies below 1 Hz result in excessively long measurement times, while frequencies above 10 Hz result in non-isothermal effects. The limits chosen are the result of extensive preliminary investigations and represent a compromise between the shortest possible measurement time and the maximum possible measurement values under isothermal conditions.

#### 2.4.2. Frequency Sweep

The amplitude sweeps were followed by frequency sweeps, for which the essential parameter settings are given in Table 4. For the Cox–Merz rule, the frequency sweeps must be performed in the LVE region. Based on the results of the amplitude sweeps, three strains were selected for the frequency sweeps.

#### 2.4.3. Ramp Test

Since conventional RPAs can only approach a limited shear rate range due to the limitation of the deflection angle to 360°, the drive unit of the RPA used in this work was modified to allow not only oscillatory movements but also continuous rotation without limitation of the deflection angle. This allows a theoretical shear rate range of up to about 750 s^−1^ to be covered, allowing viscosity data to be determined over several decades for a practical, relevant shear rate range.

To determine the steady-state shear viscosity, the lower half of the test chamber rotates with a specified speed, while the torque required to deform the material specimen is measured at the upper half of the test chamber. The resulting shear rate is given by Equation (12):

(12) $$\dot{\gamma }=\frac{v}{H_{RPA}}=\frac{U_{RPA}\times n_{0}}{2\times \left(\tan\left(\frac{\alpha }{2}\right)\times R_{RPA}\right)}$$

 v: Peripheral speed
$H_{RPA}$: Die gap
$U_{RPA}$: Test-chamber perimeter
$n_{0}$: Rotational speed
$R_{RPA}$: Test-chamber radius

The resulting edge shear stress is described by Equation (13), considering the measured torque [13]:

(13) $$\tau_{edge}=\frac{3\times M}{\pi \times {R_{RPA}}^{2}}$$

 M: Torque

The quotient of edge shear stress and shear rate results in the corresponding shear viscosity. Depending on whether the evaluation is performed in the transient or steady-state range, the result will be either the transient shear viscosity or the steady-state shear viscosity. The steady-state shear viscosity is used in the following section to compare the viscosity data with that of the HPCR. The geometric design of the test chamber with radial grooves assumes that wall slip effects are prevented [1] and the true steady-state shear viscosity is determined directly.

During the investigations, shear rates in the range of 0.1 s^−1^ to 20 s^−1^ were applied. Lower shear rates could not be achieved due to an inadequate noise-to-measurement signal ratio. Higher shear rates resulted in pronounced non-isothermal effects for the filled rubber compounds. Higher shear rates lead to a pronounced heating of the filled rubber compounds in the case of wall adhesion, despite activation of the factory-installed compressed air cooling, so that the boundary condition of an isothermal measurement is not given. Using the EPDM compound Pr. 3, the degree of heating at shear rates of 50 s^−1^ and 100 s^−1^ during a measurement time of 60 s was investigated. While forced air cooling limits the material heating to about 8 °C at a shear rate of 50 s^−1^, doubling the shear rate results in a temperature increase of about 30 °C. Since the heating of the material starts immediately at the beginning of the shear, it is not possible to reach the steady shear range with shorter measurement times, which is why a shear rate of 20 s^−1^ represents the limit for isothermal measurements.

The shear rates were applied sequentially in a single measurement. Preliminary investigations showed that separate measurements, with only one shear rate applied at a time, had a negligible effect on the resulting viscosity data while increasing the time required for the measurements. The use of a release film was avoided in this series of investigations because the continuous rotation would destroy the release film. The measurement procedure for the ramp tests is summarized in Table 5. Between each shear rate step, the material sample was held for one minute without deformation. Due to the radial grooves of the plates, it was initially assumed that slippage was prevented and that the true steady-state shear viscosity was obtained directly.

## 3. Results

In a previous study [2], the procedure as well as the evaluation routines of rheological investigations by means of HPCR analysis were described in detail. For this reason, this will not be a part of this work. In the following section, the applicability of the Cox–Merz rule and the ramp tests for the RPA used in this work will first be verified using LDPE. Subsequently, the results for both the raw EPDM polymer and the filled EPDM compounds are presented and discussed.

### 3.1. Lupolen 1840D

**Figure 5:**
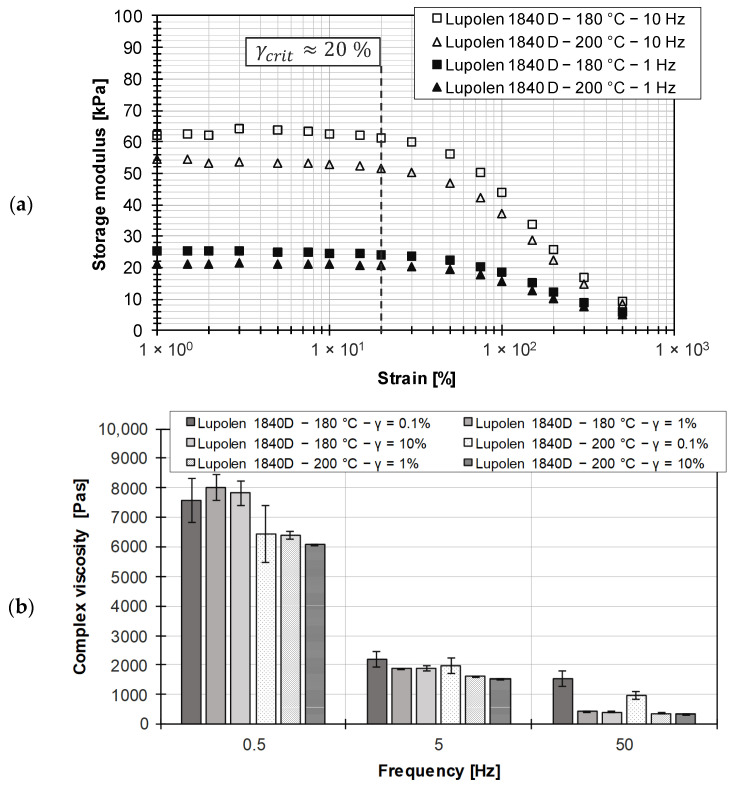
(**a**) Determination of LVE region for LDPE; (**b**) comparison of complex viscosity for different frequencies at different strains for LDPE.

In order to demonstrate the applicability of the Cox–Merz rule and the ramp test method for the RPA used in this work, preliminary tests were performed on a non-additivated low-density polyethylene (Lupolen 1840 D, LyondellBasell (Rotterdam, The Netherlands)). In Figure 5a, the frequency-dependent amplitude sweep is shown for two temperatures within the material processing range. A pronounced LVE region is identifiable, with the frequency-independent limit located at about 20% strain. Figure 5b compares the resulting complex viscosities at three frequencies over two decades. It can be seen that the values at 0.1% strain are shifted to slightly higher values than at higher strains due to the ratio of noise to measured signal. Nevertheless, it can be stated that within the LVE region there is no frequency dependence of the complex viscosity, while a higher processing temperature leads to a higher mobility of the molecular chains and thus to a reduction in the complex viscosity.

**Figure 6:**
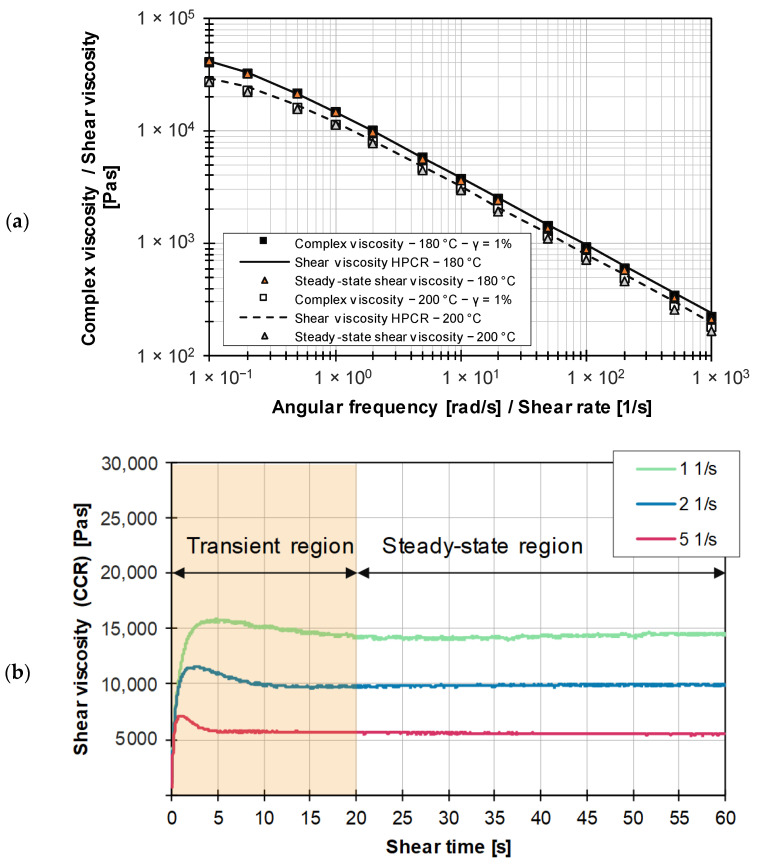
(**a**) Comparison of viscosity data based on different methods for LDPE; (**b**) differentiation between transient and steady-state shear viscosity.

Figure 6a shows the comparison between the complex viscosity based on frequency sweeps and the shear viscosity from HPCR measurements using the Cox–Merz rule. The graphs represent the measured data approximated by the Carreau model (see Equation (15)), the parameters of which are summarized in Table 6. It is noted that the Cox–Merz relationship is valid for the unfilled thermoplastic. Accordingly, it is shown that the RPA used, as well as the associated frequency sweeps, are suitable for proving the Cox–Merz rule, which provides a starting point for the investigations on practical EPDM compounds.

Figure 6b shows the time history of the viscosity using ramp tests. A stability criterion must be defined in order to distinguish between the transient and steady-state regions in ramp tests. Since the transient region depends on the shear rate, a sufficient measuring time must be provided in order to be able to make a statement about the steady-state shear viscosity. For the steady-state (time-invariant) measurement range, it is assumed that a maximum permissible deviation of 0.5% of the measurement data must be maintained for a time interval of 10 s and a measurement time of 60 s.

Under isothermal and wall-adhering conditions, the viscosity is not a function of the test geometry and is therefore independent of the rheometer used. Accordingly, the steady-state viscosity data obtained from ramp tests and the viscosity values generated by HPCR must be identical. To ensure this, an adjustment of Equation (13) for the calculation of the edge shear stress in the form below is required based on the RPA used in this work, resulting in Equation (14):

(14) $$\tau_{edge}=\frac{M}{\pi \times {R_{RPA}}^{2}}\times \left(3+\frac{1}{2}\times \frac{\log\left(M_{2}\right)-\log\left(M_{1}\right)}{\log\left({\dot{\gamma }}_{2}\right)-\log\left({\dot{\gamma }}_{1}\right))}\right)$$

Visual comparison of the steady-state shear viscosity with the viscosity data obtained from the rheological measurements using the HPCR indicates that the results are in good agreement (see Figure 6a). To characterize the rheological data for a given reference temperature $T_{b}$, the tri-parametric Carreau approach is used in combination with the temperature shift factor $a_{T}$ according to Equation (15) [3]:

(15) $$\eta =\frac{A\times a_{T}}{{\left(1+B\times \dot{\gamma }\times a_{T}\right)}^{C}}$$

A: Zero shear viscosity
$a_{T}$: Temperature shift factor
B: Transition time
C: Flow exponent

Table 6 summarizes the Carreau parameters for the HPCR, Cox–Merz, and ramp test viscosity curves. The data emphasize that comparable viscosity data are obtained for the unfilled LDPE using the procedures and methods presented.

### 3.2. Keltan 6950C

**Figure 7:**
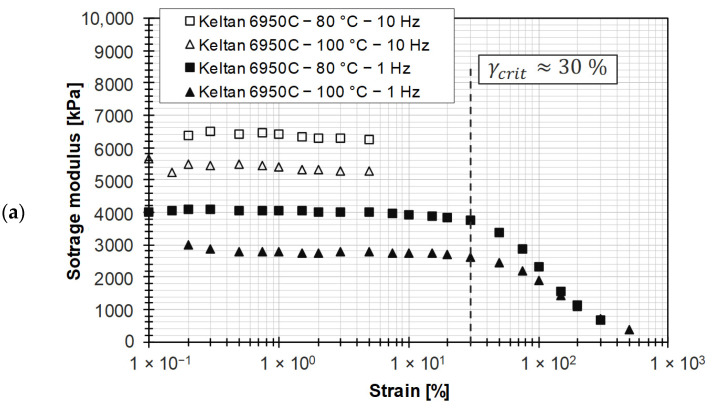

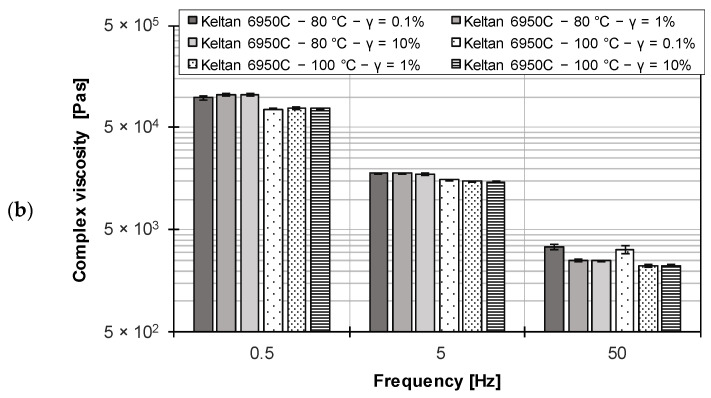
(**a**) Determination of the LVE region for the EPDM raw polymer; (**b**) comparison of complex viscosity for different frequencies at different strains for EPDM raw polymer.

In Figure 7a, the storage modulus is plotted for two frequencies and test temperatures as a function of strain for the raw polymer used in the EPDM compounds. It is observed that the EPDM raw polymer exhibits a pronounced LVE region up to about 30% strain at a frequency of 1 Hz. At a frequency of 10 Hz, higher strains resulted in pronounced non-isothermal effects due to dissipative material heating, so these are also not considered for the filled rubber compounds. Figure 7b shows that the test temperature has almost no effect on the flow behavior. Furthermore, a variation of the elongation in the LVE region does not lead to an influence on the resulting complex viscosity.

**Figure 8:**
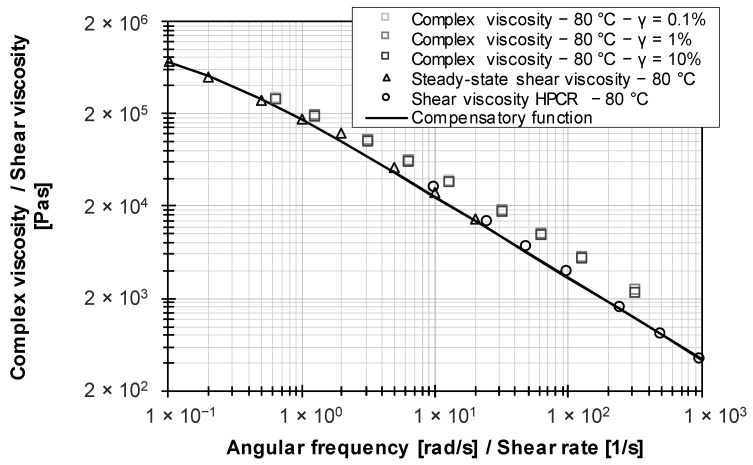
Comparison of viscosity data for EPDM raw polymer using different methods.

Contrary to the literature [32,33], where the complex viscosity from frequency sweeps correlates with the steady-state shear viscosity, the Cox–Merz relationship could not be verified for the studied EPDM raw polymer (see Figure 8). Buhrin and Rauschmann [32] explain this by referring to the challenges of HPCR measurements on unfilled rubber polymers.

However, dynamic frequency tests are intended to replace time-consuming HPCR measurements, which is why a shift factor according to Gleißle and Hochstein (see Equation (8)) is not known. Compared to the Cox–Merz relationship, the steady-state shear viscosity method allows the determination of rheological data that are in agreement with those obtained from HPCR measurements, as indicated by a compensatory function.

### 3.3. Rubber Compound with Wall-Adhering Properties (EPDM Pr. 5)

**Figure 9:**
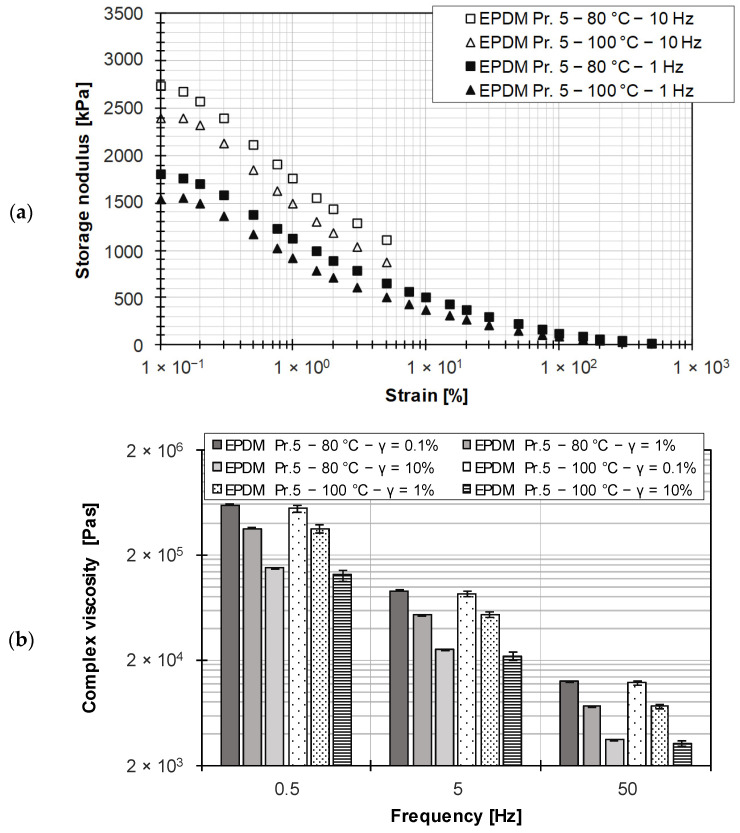
(**a**) Determination of the LVE region for the silica-filled EPDM rubber compound; (**b**) comparison of complex viscosity for different frequencies at different strains for the silica-filled EPDM rubber compound.

Compared to unfilled polymers, filled rubber compounds exhibit a more pronounced dependence of the storage modulus on strain, since it is primarily the unstable filler–filler network that gradually breaks down under stress. This phenomenon is known as the Payne effect [25] and is illustrated in Figure 9a for the wall-adhering EPDM rubber compound. The wall adhesion of this rubber compound was demonstrated in the HPCR measurements based on the geometry independence of the viscosity data over a practical shear rate range of three decades.

From the amplitude sweep data, it is not possible to determine the limit value of the LVE region for the filled rubber compound because the storage modulus decreases steadily over the strain range considered. However, since the applicability of the Cox–Merz rule requires performing a frequency sweep in the LVE region, the results of the frequency sweep at three different strains over two decades are shown in Figure 9b. It is observed that outside the LVE region, there is a significant dependence of the complex viscosity on the set strain at constant frequency, such that the complex viscosity is shifted to lower values with increasing strain. For statistical verification, the investigations were performed three times.

**Figure 10:**
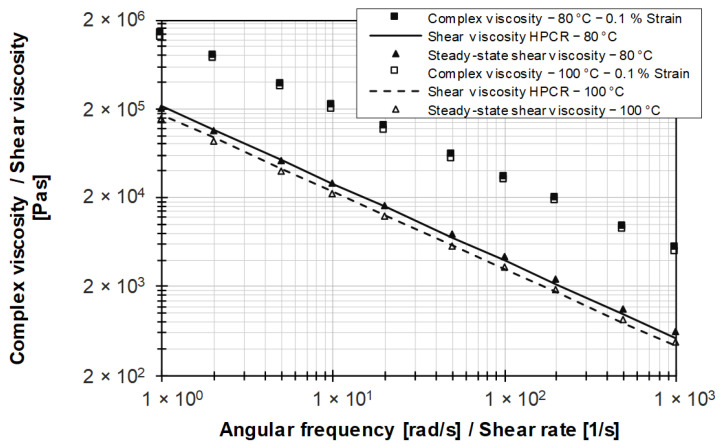
Comparison of viscosity data based for the silica-filled EPDM rubber compound using different methods.

Since the LVE region is outside the measurement range and thus shifted to lower measurement values, the complex viscosity based on 0.1% strain is considered in the following. Figure 10 shows that the Cox–Merz rule is not valid for the filled rubber systems, as the complex viscosity values are shifted to higher values by about a decade compared to those in the corrected HPCR data. This confirms the results of Hornig and Kielmann [22] for filled HNBR compounds and of Rauschmann [33] for an EPDM compound. Norton and Isayev [44] have shown this for carbon black-filled systems based on rubber polymers covering a wide range of chemical structures and molecular weights.

In contrast, the ramp test method allows for the determination of viscosity data that are in good agreement with the corrected HPCR data. Since viscosity is a rheological and material-specific parameter, it is independent of the test geometry used in the case of wall adhesion [16,18]. Extensive studies using the HPCR have shown no geometry dependence of the flow or viscosity curve, so that wall slip effects can be neglected.

Table 7 summarizes the power law parameters for each method, which, on the one hand, disprove the validity of the Cox–Merz rule and, on the other hand, show the correlation between the corrected viscosity data from the HPCR measurements and those of the RPA measurements based on ramp tests.

### 3.4. Rubber Compound with Wall-Slipping Properties (EPDM Pr. 3)

**Figure 11:**
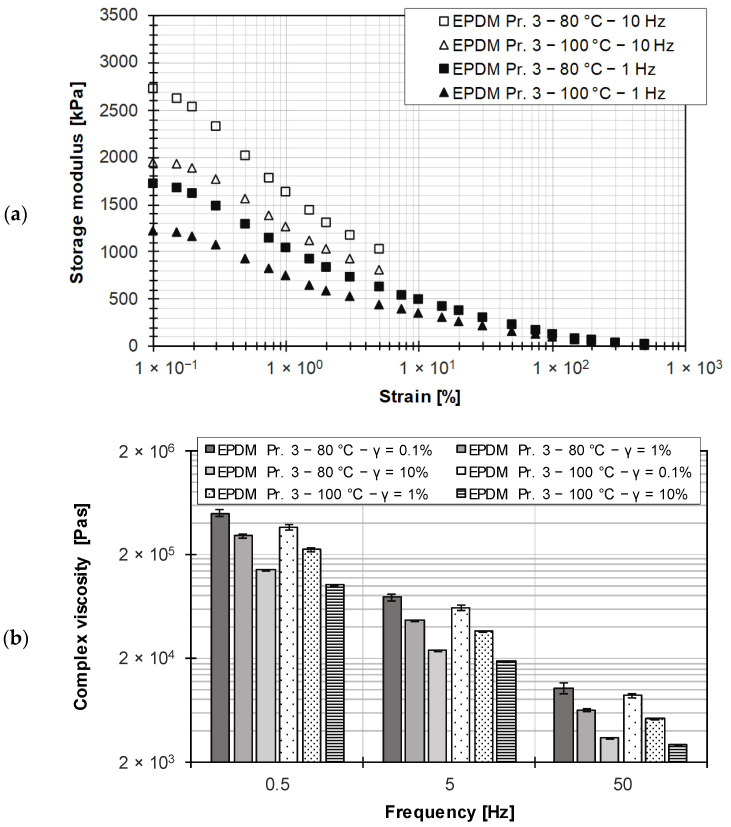
(**a**) Determination of the LVE region for the carbon black-filled EPDM rubber compound; (**b**) comparison of complex viscosity for different frequencies at different strains for the carbon black-filled EPDM rubber compound.

In addition to a rubber compound adjusted for wall adhesion by the use of silica, a rubber compound loaded with carbon black was also investigated. With this compound, pronounced wall slip effects were demonstrated in the HPCR measurements and in the extrusion process [2]. Similar to the silica-filled rubber compound, no LVE region could be detected for the carbon black-filled rubber compound (see Figure 11a), since the LVE region lies outside the measurement range in which no reproducible measurement data could be determined due to the insufficient noise-to-measurement signal ratio. Performing frequency sweeps in a strain range of the nonlinear viscoelastic region (LAOS) results in a dependence of the complex viscosity on the applied strain (see Figure 11b). For statistical verification, the investigations were performed three times.

**Figure 12:**
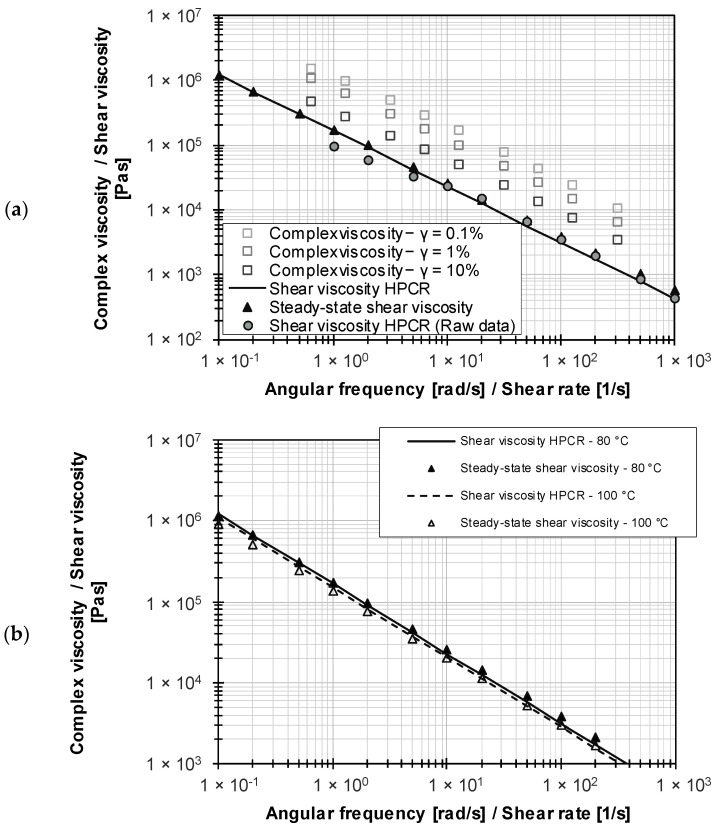
(**a**) Comparison of the complex viscosity and shear viscosity regarding uncorrected data from HCPR measurements of a carbon black-filled EPDM rubber compound; (**b**) comparison of complex viscosity and shear viscosity regarding corrected data from HCPR measurements for the carbon black-filled EPDM rubber compound.

Figure 12a compares the uncorrected (raw data) and the fully corrected HPCR data with the complex viscosity data from frequency sweeps (LAOS) and from the ramp test. As noted above, frequency sweeps outside the LVE region led to strain-dependent results with respect to the complex viscosity. A variation of the Cox–Merz rule using a shift factor according to Equation (8) is only possible if the HPCR data are available, so no general validity can be assumed without the material-specific proof of the Cox–Merz relationship.

In comparison, the use of ramp tests allows the determination of viscosity data that correlate with the corrected HPCR data (see Figure 12b). In the case of wall slip effects, there is no correlation with the ramp test data (see Figure 12a). This provides evidence that the use of double-sided engagement flanks, in combination with the current internal chamber pressure, results in wall adhesion in the RPA where wall slip corrections are not required compared to those in the HPCR.

Table 8 summarizes the power law parameters for the carbon black-filled EPDM compound, demonstrating the invalidity of the Cox–Merz rule as well as the transferability of the corrected viscosity data from the HPCR and those from the RPA based on ramp tests.

## 4. Conclusions and Outlook

The aim of this study was to investigate possible alternatives to the conventional determination of viscosity data collected with an HPCR by using an RPA. The Cox–Merz relationship was investigated as an empirical transfer function and could not be verified for an unfilled rubber polymer or for filled rubber compounds. As a result, the Cox–Merz relationship does not have unlimited validity for polymer melts and elastomers, which is consistent with previous findings. In contrast, ramp tests are a promising option for obtaining viscosity data under wall-adherent and isothermal boundary conditions. While HPCR allows a broader realizable shear rate range, no time-consuming correction procedures or cleaning efforts are required. Deviations in viscosity data between HPCR and ramp test measurements can be attributed to wall slip effects and non-isothermal effects due to dissipative material heating, while the correction for dissipative material heating and wall slip effects provides good agreement with ramp test data. Further studies will investigate the applicability of ramp tests to tire tread compounds.

## Figures and Tables

**Table 1:** Ingredients of the used rubber compounds and associated Mooney viscosity.

Component	EPDM Compound Pr. 3	EPDM Compound Pr. 5
Raw polymer (Keltan 6950 C)	100 phr	100 phr
Carbon black (N 550)	100 phr	65 phr
Silica	-	25 phr
Other fillers	20 phr	90 phr
Oil	50 phr	55 phr
Other additives	14.25 phr	14.25 phr
Total amount	284.25 phr	349.25 phr
Mooney viscosity ML_1+4_ (100 °C)	76 MU	85 MU

**Table 2:** Main specifications of the used RPA.

Specification	Specification Value	
Die gap	0.50 mm	
Frequency range	0.001 Hz to 100 Hz	
Strain	Oscillation	±0.001° to ±360°
	Continuous rotation	No limitation
Torque range	0.0001 dNm to 235 dNm	
Temperature range	Ambient to 350 °C	

**Table 3:** Test parameters for the amplitude sweeps.

	Parameter	Parameter Setting	
Preconditioning	Frequency	0 Hz	
	Strain	0%	
	Time	5 min	
	Temperature	LDPE	180 °C/200 °C
		EPDM	80 °C/100 °C
Amplitude sweep	Frequency	1 Hz/10 Hz	
	Strain	±0.1% to 500% in 23 steps	
	Number of cycles	10 cycles per strain	
	Temperature	LDPE	180 °C/200 °C
		EPDM	80 °C/100 °C

**Table 4:** Test parameters of the frequency sweeps.

	Parameter	Parameter Setting	
Preconditioning	Frequency	0 Hz	
	Strain	0%	
	Time	5 min	
	Temperature	LDPE	180 °C/200 °C
		EPDM	80 °C/100 °C
Frequency sweep	Frequency	0.1 Hz to 50 Hz in 9 steps	
	Strain	0.1%/1%/10%	
	Number of cycles	10 cycles per frequency	
	Temperature	LDPE	180 °C/200 °C
		EPDM	80 °C/100 °C

**Table 5:** Test parameters of the ramp tests.

	Parameter	Parameter Setting	
Preconditioning	Frequency	0 Hz	
	Strain	0%	
	Time	5 min	
	Temperature	LDPE	180 °C/200 °C
		EPDM	80 °C/100 °C
Continuous rotation	Shear rate steps	0.1 s^−1^ to 20 s^−1^ in 8 steps	
	Shear time per shear rate	1 min	
	Holding time between shear rate steps (No material deformation)	1 min	
	Temperature	LDPE	180 °C/200 °C
		EPDM	80 °C/100 °C

**Table 6:** Comparison of the Carreau parameters of different approaches for the investigated LDPE.

Carreau Parameter	Shear Viscosity (HPCR)		Complex Viscosity (RPA)		Steady-State Shear Viscosity (RPA)	
$T_{b}$ [°C]	180	200	180	200	180	200
A [Pas]	64,676.5	40,265.3	58,455.8	34,122.3	61,913.7	34,318.0
B [s]	10.49	6.53	8.56	4.99	9.20	5.10
C [-]	0.60	0.60	0.61	0.61	0.62	0.62

**Table 7:** Comparison of the power law parameters of the different approaches for the investigated wall-adhering EPDM rubber compound.

Power Law Parameter	Shear Viscosity (HPCR)		Complex Viscosity (RPA, 0.1% Strain)		Steady-State Shear Viscosity (RPA)	
$T_{b}$ [°C]	80	100	80	100	80	100
K [Pas]	214,404.54	170,912	1,437,150.1	1,286,695.6	205,404.29	155,354.83
n [-]	0.13	0.13	0.19	0.19	0.16	0.16

**Table 8:** Comparison of the power law parameter of the different approaches for the investigated wall-slipping EPDM rubber compound.

Power Law Parameter	Shear Viscosity (HPCR)		Complex Viscosity (RPA, 0.1% Strain)		Steady-State Shear Viscosity (RPA)	
$T_{b}$ [°C]	80	100	80	100	80	100
K [Pas]	166,478.8	149,267.56	1,138,757.9	875,976.1	170,912.7	132,706.9
n [-]	0.14	0.14	0.21	0.21	0.17	0.17

## Data Availability

The data that support the findings of this study are available on request from the corresponding author.
